# Serum response factor, a novel early diagnostic biomarker of acute kidney injury

**DOI:** 10.18632/aging.202381

**Published:** 2021-01-05

**Authors:** Long Zhao, Chenyu Li, Chen Guan, Ning Song, Hong Luan, Congjuan Luo, Wei Jiang, Quandong Bu, Yanfei Wang, Lin Che, Yan Xu

**Affiliations:** 1 Department of Nephrology, The Affiliated Hospital of Qingdao University, Qingdao 266003, China; 2 Department of Obstetrics, Weifang People’s Hospital, Weifang 261041, China

**Keywords:** acute kidney injury, biomarker, serum response factor

## Abstract

Objective: Studies have shown that serum response factor (SRF) is increased in chronic kidney injury, such as diabetic nephropathy, hyperuricemic nephropathy and renal cell carcinoma. The objective is to explore the early diagnostic value of SRF in acute kidney injury (AKI).

Methods: AKI-related microarray data were analyzed, and the expression and location of SRF were investigated in the early phase of AKI.

Results: Bioinformatics results demonstrated that SRF was dramatically elevated 2-4 h after ischemia/reperfusion (I/R) in mouse renal tissue. In I/R rats, SRF was mostly expressed and located in renal tubular epithelial cells (TECs). SRF started to increase at 1 h, peaked at 3-9 h and started to decrease at 12 h after I/R. The areas under the ROC curve of renal SRF mRNA, renal SRF protein, urinary SRF, serum SRF and serum creatinine (Scr) were 87.9%, 83.0%, 81.3%, 78.8%, 68.8%, respectively.

Conclusion: SRF is remarkably upregulated in early (before 24 h) AKI and can replace Scr as a potential new early diagnostic biomarker of AKI.

## INTRODUCTION

According to KDIGO Clinical Practice Guideline for Acute Kidney Injury [1], acute kidney injury (AKI) is defined as any of the following: increase in Scr by ≥0.3 mg/dl (≥26.5 μmol/l) within 48 hours, or increase in Scr to ≥1.5 times baseline, which is known or presumed to have occurred within the prior 7 days, or urine volume <0.5 ml/kg/h for 6 hours. AKI is a common complication and an important cause of death in critically ill patients. It is also a major factor leading to multiple organ failure. The average mortality rate is as high as 50%-65.2%. The rate of survivors progressing to chronic renal failure is as high as 35.8%-58%, and 7.5% of patients require maintenance dialysis [2]. In recent years, studies have shown that renal tubular epithelial cells (TECs) are the earliest lesion in AKI, presenting various forms of cell injury and apoptosis, while the recovery of renal function depends on surviving TECs, which can reconstruct renal tubules by dedifferentiation and proliferation [3]. Therefore, it is of great clinical significance for the prevention and treatment of AKI to further explore the mechanism of TEC injury and to search for early TEC injury markers.

Clinically, renal ischemia/reperfusion (I/R) injury is a common cause of AKI [4] and one of the major factors affecting early functional recovery and long-term survival after kidney transplantation [5]. Transient I/R causes TECs polarity loss; prolonged ischemia results in irreversible TEC necrosis or apoptosis. However, so far, there are neither reliable early markers for the diagnosis of AKI nor effective drugs for the prevention or treatment of AKI.

Currently, blood urea nitrogen (BUN), serum creatinine (Scr) and some biomarkers are regarded as diagnostic criteria for AKI. Nevertheless, all these markers lack specificity and sensitivity, causing inevitable hysteresis. Therefore, they cannot reveal the ultra-early phase of AKI [6]. Hence, to intervene in AKI earlier, an ultra-early biomarker is of urgent importance to diagnose AKI.

As an extremely conserved transcription factor of the MADS-box trans-acting factor family, serum response factor (SRF) plays a major role in the cytoskeleton and in contractile gene transcription in most cells in the vast majority of species [7]. By acting with cofactors, SRF activates immediate early genes such as c-fos [8] and Cyr61 [9] to respond to specific stimuli. Interestingly, both c-fos [10] and Cyr61 [11] were highly expressed in early AKI. SRF is crucial to the maintenance of renal TEC morphology by regulating actin cytoskeleton; moreover, SRF knockout in mouse podocytes led to foot process disappearance and chronic renal failure, suggesting that SRF is critical for kidney function and is likely to play a role in AKI. Our previous studies showed that the SRF level of TECs was upregulated in chronic kidney injury, such as diabetic nephropathy [12], hyperuricemic nephropathy [13] and renal cell carcinoma. In addition, it has been shown that SRF is increased in fibrosis and other injury conditions [14]. Nevertheless, the regulation and function of SRF in AKI remain unknown.

Gene microarrays are a proven technique that play a critical role in present medical studies [15]. The entire transcriptome can be analyzed using cDNA chips, including differences in mRNA expression of various kinds of cells. Gene microarrays are a more accurate and comprehensive study of early biomarkers and of the pathogenesis and treatment of diseases. In the present research, public database microarray data associated with AKI were analyzed, and the expression and location of SRF were investigated in the early phase of AKI. In addition, SRF expression in kidney, urine and serum was also analyzed in I/R rats, verifying the validity of the gene microarray data. Furthermore, the SRF level was compared to the Scr level to investigate the diagnostic potential of SRF in early AKI.

## RESULTS

### The microarray analysis results of bilateral I/R mice

**Figure 1:**
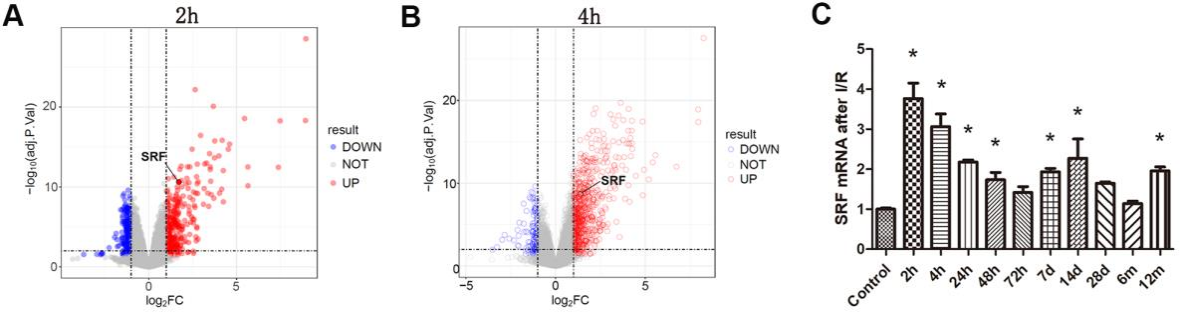
**Analysis of the microarray results from bilateral I/R mice.** (**A**, **B**) Gene expression profiling of kidney at 2 h and 4 h after I/R compared with the control group. Volcano plot of log_2_ fold changes versus -log_10_ FDR showed transcriptional differences between the I/R group and the control group. Vertical dashed lines denote the cutoff of 2-fold change, and the horizontal dashed line represents the 0.05 FDR cutoff. SRF was increased after 2 h renal I/R in mice (fold change>2, FDR<0.05). (**C**) SRF expression in I/R kidneys at 2 h, 4 h, 24 h, 48 h, 72 h, 7 d, 14 d, 28 d, 6 m and 12 m. Data are expressed as the mean ± SE. *FDR<0.05 (linear models for microarray analysis) versus the control group.

There were 567 DEGs at 2 h and 857 DEGs at 4 h after I/R in GSE98622 (Figure 1A, 1B). SRF started to increase at 2 h-4 h and decreased to the basal level at 72 h after bilateral I/R in mice (Figure 1C), which suggested that SRF might be connected with AKI. Moreover, as shown in Figure 1C, SRF was increased again 7 d after I/R, which indicated that SRF might be a promising biomarker of the transformation from AKI to chronic kidney injury and post-AKI fibrosis.

### SRF was upregulated and localized in TECs in I/R rats

**Figure 2:**
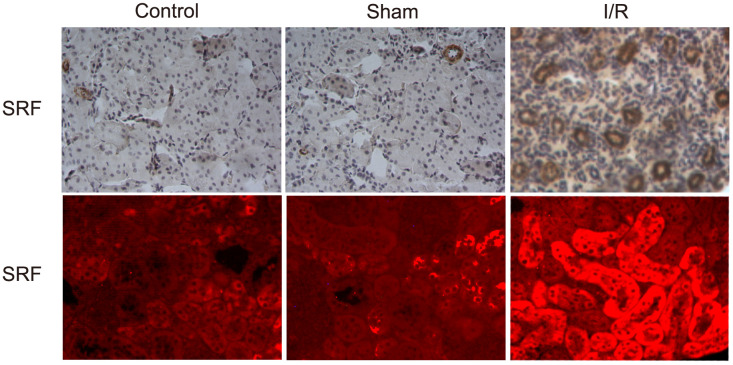
**SRF was upregulated and localized in TECs in I/R rats.** Location of SRF in kidney 6 h after I/R as evidenced by IHC and IF staining. Data are representative of at least three separate experiments.

As shown by IHC and IF, SRF was upregulated and located in TECs 6 h after I/R compared to the sham operation group and the control group (Figure 2). This result was consistent with the microarray data from I/R mice.

### Scr level and renal SRF mRNA expression in I/R rats

**Figure 3:**
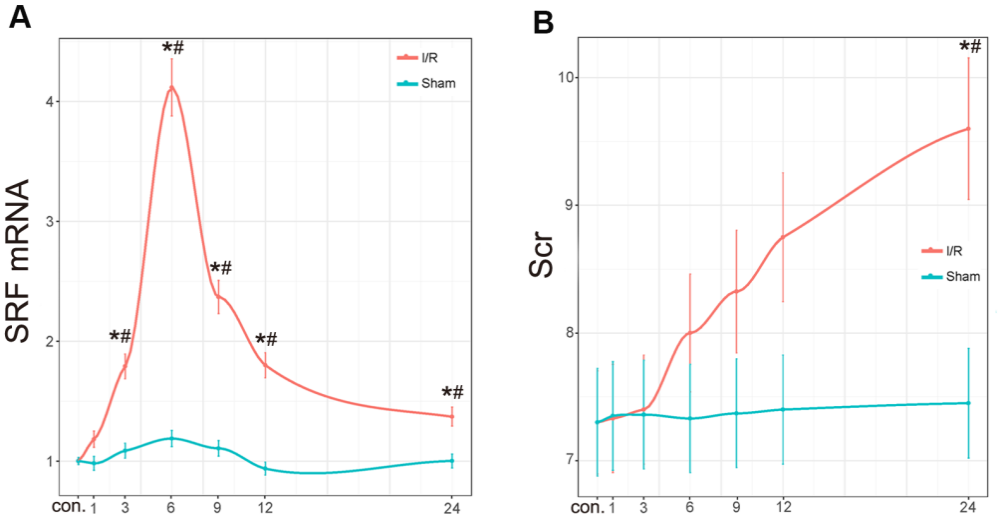
**Scr level and renal SRF mRNA expression in I/R rats.** (**A**) mRNA expression of SRF in I/R kidneys. The trend line was calculated by local polynomial regression fitting. (**B**) Scr levels in I/R rats. Data are expressed as the mean ± SE (N=7 in each group). *P<0.05 versus the sham group, ^#^*P*<0.05 versus the control group. Data are representative of at least three separate experiments.

As shown in Figure 3A, the expression of SRF mRNA in the kidney changed 1 h, 3 h, 6 h, 9 h, 12 h, and 24 h after I/R. In other words, the SRF level in the kidney of the I/R rats started to increase after 3 h, reached its peak at 6-9 h and decreased at 12 h and 24 h. The fitting function estimated that the peak SRF level should be at 6 h. No significant variation in SRF was detected in the sham operation group (Figure 3A). Compared to the increase in SRF levels, the increase in Scr levels was obviously delayed. Scr started to increase after 24 h in the I/R group compared to the control group and the sham group (Figure 3B), indicating that SRF may be an earlier biomarker for AKI and may assist physicians in diagnosing AKI in a timelier manner.

### The protein level of SRF in kidney, urine and serum *in vivo*

**Figure 4:**
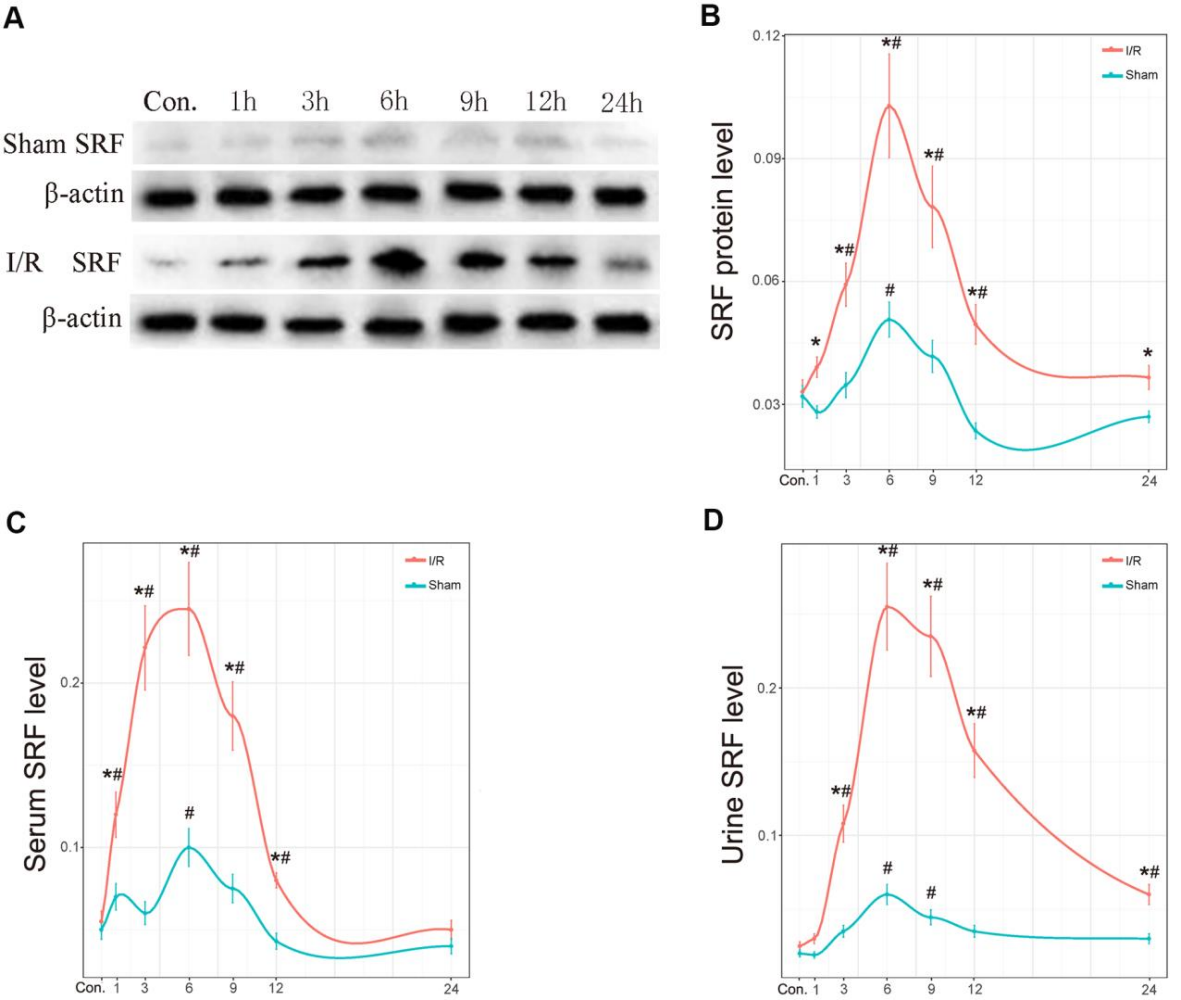
**The protein level of SRF in kidney, urine and serum *in vivo*.** (**A**, **B**) Protein expression level of SRF in kidney as measured by western blot and quantitative analyses. (**C**) The expression of SRF in serum. (**D**) The expression of SRF in urine. The trend line was calculated by local polynomial regression fitting. Data are expressed as the mean ± SE (N=7 in each group). *P<0.05 versus the sham group, ^#^*P*<0.05 versus the control group.

As shown by western blot analysis and ELISA, SRF levels in the kidney increased at 1 h, reached its peak at 6 h and started to decline at 12 h after I/R (Figure 4A, 4B). SRF was dramatically increased in the serum of I/R rats at 1 h-6 h after I/R (Figure 4C). In urine, the expression of SRF in the I/R group was dramatically increased at 3 h, reached its peak at 6-9 h, and began to decrease at 12 h after I/R. Additionally, SRF levels were also increased postoperatively in the kidney, urine and serum of the sham rats, but the increases in the sham group were significantly less than those in the I/R group.

To investigate whether SRF correlated with renal injury severity, we added 4 groups (15min, 30min, 45min and 60min renal pedicles occlusion) to study the correlation between SRF and kidney injury. As shown in Supplementary Figure 1, SRF was positively correlated with the renal injury, the expression of SRF mRNA and protein was elevated with the extension of renal pedicles occlusion time.

### The ROC curve of renal SRF mRNA, renal SRF protein, urinary SRF, serum SRF and Scr in early (before 24 h) AKI

**Figure 5:**
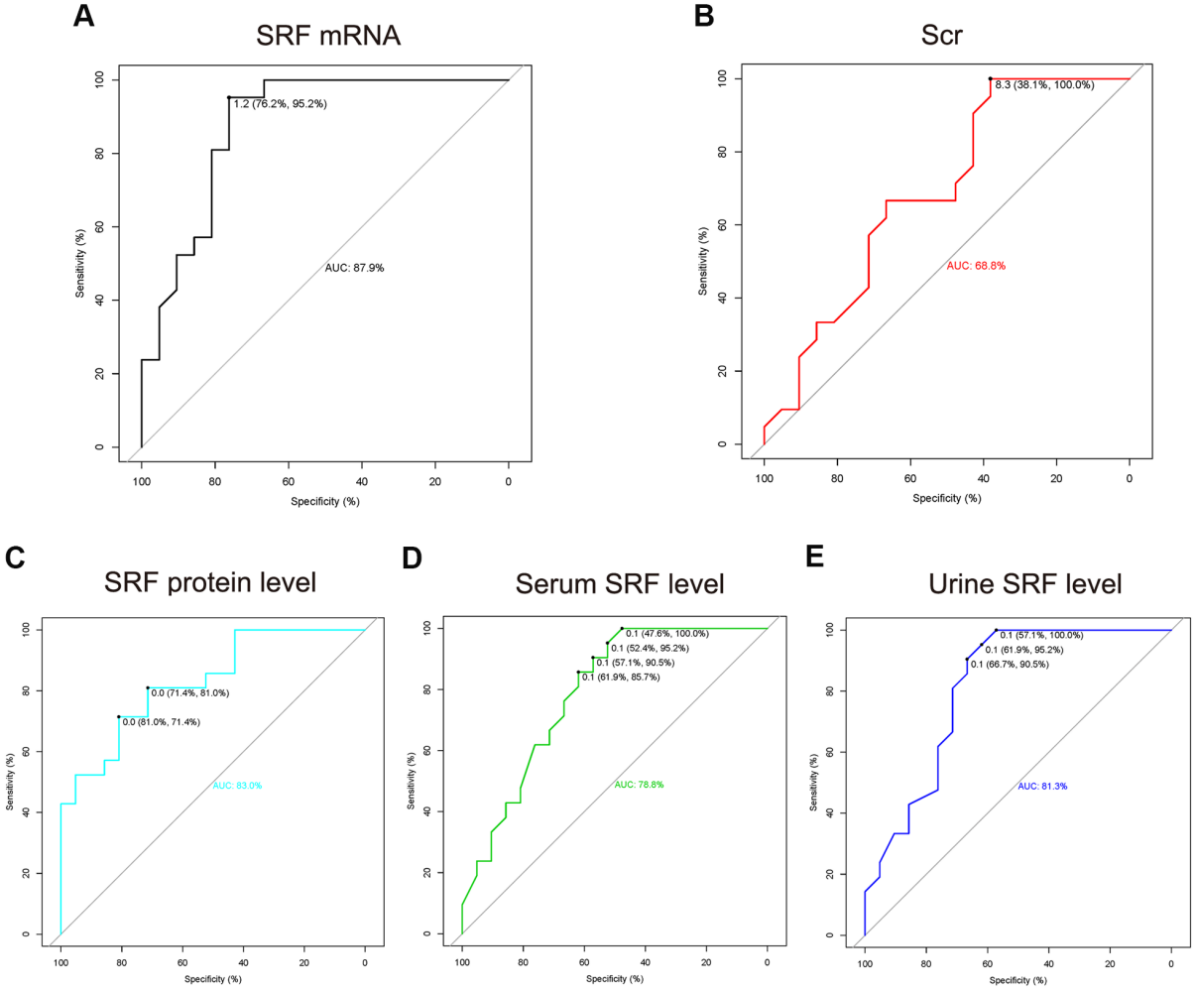
**The ROC curve of renal SRF mRNA, renal SRF protein, urinary SRF, serum SRF and Scr in early (before 24 h) AKI.** (**A**–**E**) The measured values (SRF mRNA, SRF protein, urinary SRF, serum SRF and Scr) of the AKI group (all time points after AKI as one group) were compared to those of the control group. The cutoff thresholds represent the fold change in measured values in the AKI group compared with the control group.

To explore more sensitive and specific biomarkers to diagnose AKI, we used a ROC curve assay to investigate the cutoff value of renal SRF mRNA, renal SRF protein, urinary SRF, serum SRF, and Scr levels (Figure 5). The AUCs of SRF mRNA, SRF protein, urinary SRF and serum SRF were 87.9%, 83%, 81.3% and 78.8%, respectively, which were all better than that of Scr (68.8%), which indicated that urinary SRF could be an early diagnostic marker of AKI with better sensitivity and specificity than Scr.

## DISCUSSION

At present, I/R is regarded as a critical process that may lead to AKI [16–18]. Because of the unique anatomical position and energy metabolism of TECs, they are especially vulnerable to I/R damage. Moreover, I/R injury factors are able to cause oxidative stress and endoplasmic reticulum stress [19], resulting in autophagy, apoptosis, cell necrosis and other adverse effects [20]. If not treated promptly and effectively, AKI may progress irreversibly in patients with chronic renal failure [21]. Hence, it is of great importance that AKI is diagnosed early and precisely. Therefore, this study aimed to find an extremely specific and sensitive biomarker for the early diagnosis of AKI, which is extremely important to improve the diagnosis, treatment and prognosis of AKI.

Immediate early genes are the first group of genes to be expressed after external stimulation. As a transcription factor that regulates the expression of immediate early genes, SRF may change before immediate early genes. Recent studies have shown that SRF improves the function of ischemic muscles by promoting microvascular growth and maturation through the immediate early genes Cyr61 and CCN2 [22]. In this study, bioinformatics analysis showed that the expression of SRF was dramatically increased after 2 h and returned to baseline after 72 h (Figure 1). In addition, our previous studies have shown that SRF is highly expressed in podocytes [23], TECs [12] and glomerular endothelial cells [24] in the kidney. These results indicate that SRF may be upregulated and function in AKI.

Recent studies have shown that SRF is upregulated in neuron I/R injury and plays a protective role [25]. In this study, the levels of SRF in the kidney and body fluid were measured separately to validate the screened DEGs. Fortunately, the mRNA and protein levels of SRF in the kidneys of I/R rats peaked during 6-9 h and were reduced moderately during 12-24 h (Figures 3, 4). In addition, our previous studies demonstrated that SRF is highly expressed in various kinds of kidney injury [23, 12, 13, 24], indicating that SRF may be a biomarker of AKI and may play a role in AKI.

Currently, BUN and Scr levels are considered the most popular diagnostic biomarkers of AKI. Nevertheless, it has been reported that BUN and Scr levels are not extremely sensitive and specific to AKI and showed clear hysteresis [1, 26]. ROC curve analysis was carried out to identify more sensitive and specific biomarkers of AKI. The areas under the ROC curve of renal SRF mRNA, renal SRF protein, urinary SRF, serum SRF and Scr were 87.9%, 83%, 81.3%, 78.8% and 68.8%, respectively (Figure 5). These findings were consistent with the bioinformatics results, confirming that SRF could become an ultra-early biomarker of AKI. In addition, as we can see in Figure 2C, SRF was still increased 7 d to 12 months after AKI, which indicated that SRF might be an important biomarker in the transformation from AKI to chronic kidney injury or post-AKI fibrosis.

Our results indicate that SRF may be a novel biomarker for the early diagnosis of AKI. It should be emphasized that the present study has some limitations, as it was only conducted *in vivo*. In the sham group, slight injury may have been caused by renal pedicle separation, leading to a slight increase in SRF, but the degree of SRF upregulation in the sham group was much lower than that in the I/R group. The expression of SRF in the I/R group was significantly higher than that in the sham group, which would not occur in a clinical setting. In addition, *in situ* hybridization of mRNA was not performed in the present research, so the clinical criteria and localization of SRF mRNA still need to be further studied. Finally, it is not clear what role SRF plays in AKI, and with further study, we speculate that SRF may improve AKI. In summary, our study investigated the expression of SRF in renal tissue, urine and blood in AKI. According to different methods, SRF is a novel biomarker for the early diagnosis of AKI.

## MATERIALS AND METHODS

### Acquisition and preprocessing of gene microarray data

We searched the public microarray database GEO (Gene Expression Omnibus, http://www.ncbi.nlm.nih.gov/geo/) for AKI-related samples. Raw data (CEL file) from GSE98622 [27] were downloaded from the GEO database. GSE98622 derived total kidney mRNAs from mice with bilateral I/R. The *oligo* package [28] was used to evaluate microarray quality. The robust multiarray averaging method [29] was then used to preprocess the raw data. Probes were annotated with the latest official annotations file. All bioinformatics analyses were processed and analyzed by R software (v.3.4.0).

### Differential gene screening

Linear models for microarray analysis [30] were used to identify differentially expressed genes (DEGs). A moderated t-test [30] was used to compare of the fold change in SRF between the AKI group and the control group, and FDR was calculated by using the Benjamini-Hochberg method. Fold changes >2 or <0.5 with FDR<0.05 were considered as the threshold to select DEGs.

### Animal protocols

A total of 119 male Wistar rats, 8 weeks of age and weighing 250-280 g, were purchased from the animal experiment center of Qingdao University (Qingdao, China). The rats were given free access to food and water throughout the study. After a minimum 7-d acclimation period, the rats were randomly divided into 3 groups: (1) control group (N=7); (2) sham-operated group (the animals were subjected to a similar surgical procedure without nephrectomy of the right kidney and without clamps on the renal pedicles, N=42); (3) I/R group. In the I/R group, the rats were under surgical anesthesia with pentobarbital sodium of 4 mg/kg and received a midline incision and right kidney resection. The left renal pedicles were exposed, and clamps were placed on them for 45 min. Then, the kidneys were reperfused as their color returned to red (N=42). 28 rats’ left renal pedicles were clamps for 15min, 30min, 45min and 60min respectively (each 7). All experiments were performed in accordance with the Chinese guidelines on the use and care of laboratory animals and were approved by the Laboratory Animal Welfare and Ethics Committee of the Affiliated Hospital of Qingdao University. The second and third groups were subdivided into 6 subgroups, as we set some time points to measure SRF and Scr changes during the first day after I/R. Rats were sacrificed at 1 h (N=7), 3 h (N=7), 6 h (N=7), 9 h (N=7), 12 h (N=7) or 24 h (N=7) after I/R.

### Immunohistochemistry (IHC)

Renal tissue samples were fixed in formalin and then embedded in paraffin. Sections with a thickness of 3 μm were incubated with a primary antibody against SRF (Santa Cruz, CA, USA, 1:500 dilution), followed by incubation with a horseradish peroxide-conjugated secondary antibody at 37° C for 30 min, visualization, counterstaining, dehydration, transparent and neutral gum sealing. Under 200× magnification, the cells with brown cytoplasmic granules were positive. Seven random fields were observed on each section.

### Immunofluorescence (IF) staining

The tissues were fixed in 10% neutral buffered formalin for 24 h and then embedded in paraffin. After deparaffinization, the slides were blocked in 1% BSA for 1 h and then co-incubated with a primary antibody targeting SRF (Santa Cruz, CA, USA, 1:500 dilution) at 4° C overnight. After washing off the primary antibody, the sections were incubated for 1 h at room temperature with the appropriate Alexa Fluor-conjugated secondary antibody (Zsgb, Beijing, China). The slides were viewed under a Nikon epifluorescence microscope.

### Quantitative RT-PCR

Total RNA from kidney tissue was isolated by using TRIzol (Invitrogen, Carlsbad, CA, USA). A total of 500 ng RNA from each sample was reverse transcribed to cDNA by a PrimeScript™ RT reagent kit (Takara, Otsu. Japan). Subsequently, quantitative RT-PCR was performed by the SYBR Green (Takara, Otsu, Japan) method. The primers used in this experiment are shown in Table 1. To normalize the mRNA quantity, GAPDH was used as the reference gene, and the 2^-ΔΔCT^ method was used to calculate the relative expression of mRNA.

**Table 1:** The primers of quantitative RT-PCR.

**Gene**	**Forward (5’→3’)**	**Reverse (5’→3’)**
SRF	GCACAGACCTCACGCAGA	ATGTGGCCACCCACAGTT
GAPDH	GGATTTGGTCGTATTGGG	GATGATCTTGAGGCTGTTGTC

### Western blotting

At sacrifice, urine, serum and kidney tissue were collected to extract protein, and RIPA buffer was used to suspend the samples. The homogenate was centrifuged at 10000 × g for 30 min at 4° C. Fifty micrograms of protein in the mixture was separated by 10% SDS-PAGE and transferred onto 0.45 μm PVDF membranes (Millipore, Germany), followed by a 1 h incubation with 5% nonfat dried milk in phosphate-buffered saline (PBS) at room temperature. Then, the cells were incubated with primary antibodies against SRF (Santa Cruz, CA, USA) and β-actin (Cell Signaling Technology, MA, USA) at 4° C overnight. After washing with PBS with Tween 20, the membrane was incubated with secondary antibodies. The target bands were visualized by chemiluminescence.

### Enzyme-linked immunosorbent assay (ELISA)

ELISA (R&D Systems, Minneapolis, MN, USA) was used to examine the content of SRF in urine and serum as previously described [11].

### Assessment of renal function

Scr was measured using the Jaffe colorimetric method as previously described [11].

### Statistical analysis

Data are expressed as the mean ± SE and were compared by Student’s t-test or one-way analysis of variance for different groups. *P*<0.05 was considered statistically significant. Local polynomial regression fitting was used to calculate the trend line of SRF mRNA level, SRF protein level, serum SRF, urinary SRF and Scr. A receiver operating characteristic (ROC) curve was used to analyze the sensitivity and specificity of SRF mRNA, SRF protein level, serum SRF, urinary SRF and Scr in the I/R group (all time points after I/R as one group) compared to the control group. All data were processed and analyzed by R software.

## Supplementary Material

Supplementary Figure 1
