# Supplementary material for: Serum response factor, a novel early diagnostic biomarker of acute kidney injury
Source: Aging (Albany NY). 2021 Jan 5;13(2):2885–94. doi: 10.18632/aging.202381 (PMC7880358; doi:10.18632/aging.202381)
Supplement: Supplementary Figure 1 [file aging-13-202381-s001.pdf]

SUPPLEMENTARY FIGURES

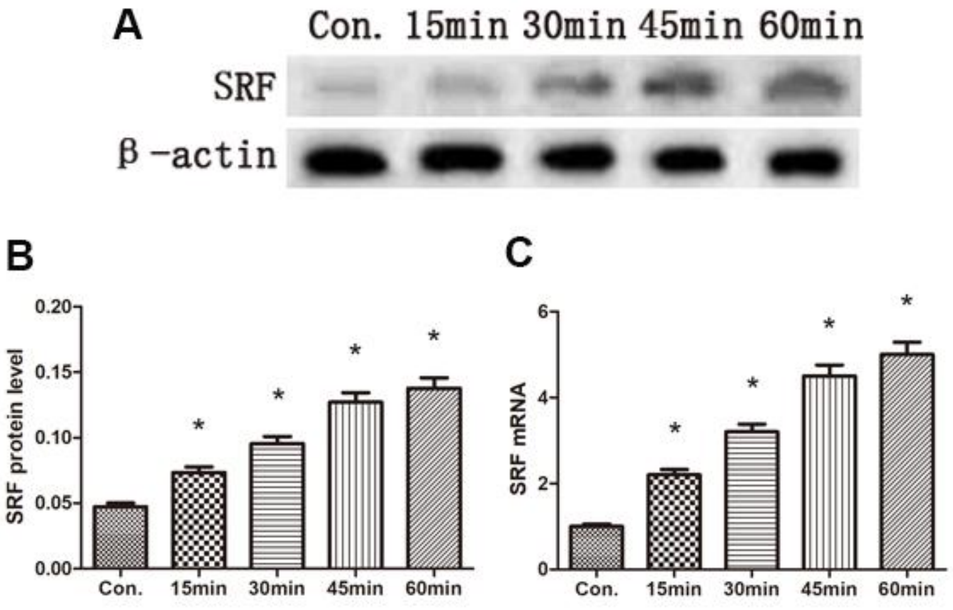

Supplementary Figure 1. SRF protein and mRNA expression was upregulated with the extension of renal pedicles occlusion time. (**A**, **B**) The protein and (**C**) mRNA expression of SRF at 6h after different renal pedicles occlusion time. Data were expressed as the mean ± SE (N = 7 rats per group). **P* <0.05 versus control group.
